# A reflection on the challenge of protecting confidentiality of participants while disseminating research results locally

**DOI:** 10.1186/s12910-018-0279-0

**Published:** 2018-06-15

**Authors:** Anne-Marie Turcotte-Tremblay, Esther Mc Sween-Cadieux

**Affiliations:** 1 University of Montreal Public Health Research Institute, Montreal, Canada; 2 University of Montreal School of Public Health, Montreal, Canada; 3 Department of Psychology, University of Montreal, Montreal, Canada

**Keywords:** Ethics, Dissemination, Confidentiality, Global Health

## Abstract

**Background:**

Researchers studying health systems in low-income countries face a myriad of ethical challenges throughout the entire research process. In this article, we discuss one of the greatest ethical challenges that we encountered during our fieldwork in West Africa: the difficulty of protecting the confidentiality of participants (or groups of participants) while locally disseminating results of health systems research to stakeholders.

**Methods:**

This reflection is based on experiences of authors involved in conducting evaluative research of interventions aimed at improving health systems in West Africa. Our observation and collaboration with the research projects’ stakeholders informed our analysis. Examples from two research projects illustrate the issues raised.

**Results:**

We found that in some cases there is a risk that local stakeholders may be able to identify research participants, or at least groups of participants, during the dissemination of results, even if they are anonymized. Four factors can interact and influence this challenge: 1) hierarchical structure, 2) small milieu, 3) immersion in a few sites, and 4) vested interests of decision-makers. For example, local stakeholders can sometimes find out when and where the data were collected. Moreover, health systems, especially rural healthcare centres, in West African countries can be small settings, so people often know each other. Some types of participants have unique characteristics or positions in the health system that may make them more easily identifiable by local stakeholders familiar with the environment. We identified a number of potential strategies that can help researchers minimize this difficulty and improve ethical research practices. These strategies pertain to the development of the study design, the process of obtaining informed consent, the dissemination of results, and the researchers’ reflexivity.

**Conclusion:**

Researchers must develop and adopt strategies that enable them to respect their promise of confidentiality while effectively disseminating sometimes sensitive results. Reflections surrounding ethical issues in global health research should be deepened to better address how to manage competing ethical responsibilities while promoting valuable research uptake.

## Background

Researchers studying health systems in low-income countries (LICs) face a myriad of ethical challenges throughout the entire research process. Distinctive features of this field of research can colour ethical issues, such as the balancing of risks and benefits for individuals, groups, and communities [1, 2]. In this article, we discuss one of the greatest ethical challenges we encountered during our fieldwork in West Africa: the difficulty of protecting the confidentiality of participants, or groups of participants, while locally disseminating results of health systems research. On one hand, researchers have to preserve the confidentiality of participants, or groups of participants, especially when the research focuses on sensitive issues such as negative or perverse effects of interventions. Breaches of confidentiality could harm participants, hinder the trust relationship between participants and researchers, and even hurt the reputation of a group or community. On the other hand, global health researchers are increasingly encouraged to disseminate research findings to local stakeholders such as decision-makers, managers, practitioners, and community members. Our experiences have sparked in us a growing concern that local stakeholders may be able to identify research participants, or at least groups of participants, during the dissemination of results. For example, local stakeholders can sometimes find out information on when and where the data were collected. In West African countries, health systems—especially rural healthcare centres—can be rather small settings, so people often know each other. Some types of participants have unique characteristics or positions in the health system that render them more easily identifiable by local stakeholders familiar with the environment. Together, such factors make it difficult to protect participants’ confidentiality during the dissemination of results locally. The objectives of this reflection are to 1) show how researchers conducting health systems research in LICs can experience difficulty in protecting the confidentiality of participants locally and 2) present some potential strategies to minimize this difficulty.

## Methods

### Background experiences

Our experiences conducting evaluative research on interventions aimed at improving health systems in West Africa have contributed to this reflection. One of our research projects was an analysis of the unintended consequences of a performance-based financing intervention in Burkina Faso. In this intervention, healthcare workers were paid for the quantity and quality of healthcare services they provided, which resulted in certain fraudulent practices being committed to increase financial gains [3]. We conducted prolonged field work in healthcare centres to collect data through observation, interviews, and discussions with participants (e.g. healthcare workers, community leaders, verifiers, healthcare users). Before leaving the host country, we wanted to disseminate the results of the study widely to local stakeholders (e.g. district management teams, representatives of the Ministry of Health and participants) while respecting ethical norms such as confidentiality. However, it became obvious that reporting research findings to local stakeholders could result in breach of confidentiality.

Our reflection was also inspired by a research project that evaluated the implementation and effects of an intervention aimed at promoting the use of research findings to influence health practices and policies in Burkina Faso. This unique intervention was implemented at the local level and only involved a few stakeholders (e.g. local and international researchers, local consultants, representatives of non-governmental organizations, community associations, and district-level decision-makers. As several difficulties arose during the intervention’s implementation process, that research shed light on what led to its failure. Thus, that study also posed some challenges in terms of identifying the stakeholders in charge of the intervention when the results were disseminated, as will be further explained below.

In hindsight, we find that our reflexive process was iterative and corresponded to the phases of Schön’s reflective practitioner model, as described by Tremblay and colleagues [4]. First, we went through the assessment phase, in which we formulated an initial understanding of a new and problematic situation, that is, the difficulty of protecting the confidentiality of participants while disseminating research results locally. In one study, for example, this issue became apparent and was explicitly discussed during the development of study protocols. Then, in the action phase, we tested this understanding and its implications in the field, for example, during data collection and dissemination of results. Lastly, in the reassessment phase, we revisited the terms of the problem, looked at it critically, and proposed solutions. The present article synthesizes this last stage.

## Results and Discussion

### An ethical responsibility to protect confidentiality

One of our major and ongoing concerns was the need to protect the confidentiality of participants.^1^ The ethical duty of confidentiality refers to researchers’ obligation to safeguard entrusted information [5, 6]. Breaches of confidentiality can have negative repercussions on participants if other stakeholders are able to identify them [7]. For example, during the dissemination of research results, if a supervisor were to discover that a specific type of health worker participating in a study engaged in prohibited behaviour, such as falsifying consultation registers, the supervisor could be tempted to take actions against them. These participants could forfeit future job opportunities, lose professional credibility, or become socially ostracized within their environment. Breaches of confidentiality could also harm the reputation of a specific community by increasing stigmatization towards them (e.g. prejudice, marginalization). As researchers, we experienced difficulties in fully grasping or anticipating the nature and amplitude of such potential repercussions, due to cultural differences and our limited understanding of the complex social structure. For example, during the risk–benefit assessment of disseminating results locally, it was difficult to determine which specific results could lead to a breach of confidentiality, to what extent such a potential breach could harm participants, and how local stakeholders could react. Thus, we feel serious consideration should be given to the protection of confidentiality upstream.

Ensuring confidentiality is also essential to build trust relationships with participants [5]. Without the assurance of confidentiality, they might refuse to share data or hide data that are important to answer research questions, especially when the study focuses on sensitive issues (e.g. hidden behaviours, controversial views, perverse effects). Lack of trust from participants could increase the risk of biases in research (e.g. social desirability).

In the context of global health research, respecting confidentiality is crucial because some participants already feel apprehensive towards researchers, who tend to be outsiders in relation to the local context. Indeed, global health researchers, whether they reside in West Africa or elsewhere, often come from different backgrounds (e.g. nationality, culture, socio-economic status)^2^ from the participants and have different mandates from each other. In our experiences, research participants were sometimes skeptical of our true affiliation and mission in their organization. Our presence was sometimes perceived as surveillance from international development agencies or program funders to evaluate an intervention. Communities who have negative experiences with an outside researcher may be less welcoming towards future researchers. In this respect, protecting confidentiality is essential to promote people’s openness towards global health researchers in participating communities and to facilitate future research.

Furthermore, power asymmetries can exist between researchers and communities as well as between communities and actors in the health system. Some participants may feel pressured to take part in a study or may unintentionally reveal sensitive information to researchers. Atchessi et al. [8] found that hierarchical authority can interfere with free and informed consent in global health research. Thus, protecting confidentiality is paramount to avoid causing undue harm to vulnerable populations who do not necessarily have sufficient means to protect their own interests. Although participatory action research may be a way to help rebalance power inequalities in research, it can lead to a range of particular ethical issues [9].

### An ethical responsibility to disseminate results

Dissemination of results is a “planned process that involves consideration of target audiences and the settings in which research findings are to be received and, where appropriate, communicating and interacting with wider policy and health service audiences in ways that will facilitate research uptake in decision-making processes and practice” [10]. The dissemination of research findings is considered to be a researcher’s ethical obligation. Ethics committees and funding agencies increasingly require that researchers conduct knowledge translation activities, including dissemination of results [2, 11, 12]. The Declaration of Helsinki stipulates that “researchers have a duty to make publicly available the results of their research on human subjects” (World Medical Association, 2013, item 36). For Canadian researchers, the Tri-Council Policy Statement [5] states that researchers should provide copies of publications and research reports to organizations that are best suited to disseminate the results within participating communities. According to that statement, this is especially important in settings where the results are not easily accessible, such as LICs. However, the definition of community is somewhat labile, so it was sometimes ambiguous for us whether we had the responsibility to disseminate results at the village, district, region, or even country level. Still, we felt our duty was to report our research results in appreciation of the community and the research participants’ involvement, and we thought our results could improve local practices and policies in health.

### Difficulties in protecting confidentiality while collaborating with local stakeholders

We found it difficult to protect the confidentiality of participants while collaborating with local stakeholders, particularly for the dissemination of results. These local stakeholders included representatives from the Ministry of Health, intervention implementers, district management teams, local leaders, participants, etc. We identified four factors that may interact and influence this challenge: 1) hierarchical structure; 2) small milieu; 3) researcher immersion in one or just a few sites; and 4) vested interests of stakeholders. These four factors emerged from our experience, but the list is not intended be exhaustive. While these factors are not ethical problems per se, they may raise ethical concerns for researchers trying to protect confidentiality while collaborating with local stakeholders. We use examples from our research experiences in West Africa to illustrate the issue.

#### Hierarchical structure

Global health researchers cannot conduct research within the health system without informing various local authorities of their presence. Both local and international researchers must conduct courtesy visits to the Ministry of Health and/or the health district offices to inform decision-makers that research will be conducted within their jurisdiction. For example, to collect data within healthcare centres, authorization has to be obtained from the person responsible for each healthcare centre in question and from authorities at the district level. These courtesy visits are important because they significantly facilitate access to the research sites. They enable superiors (e.g. chief medical officer in a district) to inform their subordinates (e.g. chief head nurses in healthcare centres) that a study will be conducted and that they can collaborate, if they wish to do so. These visits are also an important step in building collaboration between the researchers and decision-makers to increase the relevance and use of results by stimulating their interest and allowing them to provide input into research questions, methods, etc. However, during these visits, local stakeholders inevitably ask researchers what healthcare centres they plan to visit. Thus, when the results are disseminated locally, these stakeholders may be more easily able to identify research sites and participants. Even if participants’ identities are masked during results dissemination, local stakeholders may be able to infer the likely source.

#### Small milieu

Many West African countries (e.g. Benin, Burkina Faso) are relatively small in terms of population size and distances. Consequently, actors within a health system tend to know each other. To promote primary healthcare, small centres have been established across many West African countries [13]. Due to limited resources, many of these centres have only about seven workers, with distinct roles. Moreover, many roles or positions, especially at the higher levels of the health system, are distinctive or singular (e.g. director, supervisor, program planner). For example, the performance-based financing intervention we studied in West Africa only had two supervisors in the district who travelled to each healthcare centre to count the quantity of healthcare services delivered. Thus, results relevant to that aspect of the intervention could be traced back to them more easily than could more generic results relevant to a wide array of actors. In such a small milieu, staff from small healthcare centres or with distinctive roles are more easily identifiable when results are disseminated locally. Because these types of participants have access to particular information and details, it can be difficult to simply aggregate their data with the rest of the data to protect their confidentiality. Thus, we were often concerned that, even if researchers attempted to protect the participants’ confidentiality, local stakeholders who knew the context, its actors, and past events might have been able to infer the likely source. As Richards et al. [7] explain, even after protocols for anonymization are applied, “quotations, speech mannerisms and context may provide enough information for participants to be identified” by local stakeholders, and it is not always easy to predict which data will lead to identification.

#### Researcher immersion in one or few sites

Studying health systems in global health can require long immersions in the field to understand the behaviours, discourses, and obstacles that emerge in real life. Prolonged fieldwork can be useful, especially for researchers coming from a different background, to develop a more profound understanding of the local context, cultures, norms, etc. It is also useful for building relationships and trust between researchers and local actors, which lead to more authentic behaviours and discourses, thereby reducing potential biases. However, as long-term immersion in an organization (e.g. a healthcare centre) is time-consuming, it significantly limits the number of research sites a global health researcher can target to collect data. With fewer research sites, it can be easier for local stakeholders to make links between specific data collection sites or participants once the study results are disseminated.

Moreover, prolonged immersion in just a few healthcare centres means that local stakeholders, such as members of the district medical team, supervisors, and healthcare workers from other organizations, can spontaneously witness a researcher collecting data during observation sessions or interviews. Thus, they can know from whom the researcher collected data for the study. Again, it may be easier for these local stakeholders to make links between the results and participants during the dissemination of results, especially since they know the context well.

#### Vested interests of stakeholders

Local stakeholders sometimes have vested interests in promoting interventions such as performance-based financing. The salaries of those who are employed to manage or implement an intervention sometimes depend on its success. Local stakeholders may be wary of or intrigued by independent researchers who evaluate interventions, because a study’s results could influence decisions to pursue or renew an intervention’s funding. Studies on sensitive topics (e.g. unintended consequences of an intervention or the causes of its failure) may be more threatening for local stakeholders, as they may reveal hidden information. If their interests are at stake, local stakeholders may be more likely to keep track of a researcher and the data collection process as it unfolds. For example, they can seek information in their network on where data are being collected. During our own research, we found it difficult to hide a researcher’s tracks during the data collection process. Ultimately stakeholder attention renders it more difficult for the researcher to hide the identities of participants during the dissemination of results.

### Potential strategies

Beyond the usual method of anonymizing data, we found there is not enough discussion on how researchers can better protect the confidentiality of participants or groups of participants in different contexts while disseminating results to local stakeholders [14]. Based on our experience, we have identified a number of potential strategies to help researchers in this endeavour:

#### Strategies related to the study design

- Adopt a design that allows the researcher to have a sufficient number of sites to protect confidentiality, while still being able to spend enough time in each site to develop a profound understanding of the context. The design can include primary cases with longer periods of fieldwork and secondary sites with shorter stays in other regions to “muddy the waters” and avoid stigmatizing specific groups of individuals.
- For qualitative studies, nest the study within a larger study to triangulate results with other locations and cover the researcher’s data collection sites.
- For quantitative studies, include a sufficient sample size to aggregate data for subgroups of participants.

#### Strategies related to informed consent

- Discuss risks of breaching confidentiality with the participants before obtaining informed consent.
- Engage in a dialogue with participants to determine whether they are comfortable with the results—anonymized—being shared with local stakeholders. However, this may bias the results of the study if participants subsequently choose to share mainly positive results and to conceal negative results.

#### Strategies related to the dissemination of results

- Discuss the issue of confidentiality with stakeholders before the dissemination of results and have them sign a confidentiality agreement.
- Target specific stakeholders with whom to share results locally. After due ethical consideration, researchers may decide it is in the best interests of the local community and future research to avoid presenting results to specific actors (e.g. immediate supervisors of participants). Instead, they may decide to present results to higher-level stakeholders for whom it may be more difficult to make direct links between the results and the participants and who would have the ability to use the results constructively. This strategy requires an in-depth risk–benefit assessment, as it may go against best practices in ethics and knowledge translation.
- Present results in a more general manner. When in doubt, researchers can omit information that may potentially be indirectly identifying. For multiple case studies in different healthcare organizations, researchers can choose to report only cross-case analyses [15].
- Reflect on how to describe the context in a way that protects collective confidentiality but is detailed enough to consider external validity. Researchers can attempt to hide the study’s location, such as the country, district, or healthcare centre. However, preserving the anonymity of the case 1) prevents people from recollecting previous information about the case when interpreting it and 2) makes the entire case more difficult to review [15]. Contextual data are often an essential component of the analysis and interpretation [7].
- Wait for some time before disseminating results to help protect confidentiality, relying on the high mobility of stakeholders within the health system. In some cases, ongoing dissemination of results throughout the research process (e.g. in developmental evaluation) may have to be avoided to make it easier to hide the researchers’ trail, although this may go against best practices in knowledge translation.
- Collaborate with a local knowledge broker to disseminate results to stakeholders. This third party may be more apt to disseminate sensitive results in a socially acceptable manner while maintaining participants’ confidentiality. The knowledge broker could also organize dialogues and act as a mediator to address these sensitive issues.

#### Strategies related to the researchers’ reflexivity

- Discuss ethical concerns and potential strategies with other colleagues who understand the context to find a solution that is adapted to the case and meets local needs.
- To orient action, continuously evaluate the risks and benefits not only for individual participants but also for social groups. The researchers’ understanding of risks and benefits may change over the course of the study.
- Use practical judgment and reflexivity to develop strategies that are adapted to the study’s context. Codes of practice cannot replace practical judgment and reflexivity, especially in the context of qualitative research on health services [7]. Several models on reflexivity can be useful for researchers to engage in this process [4].

## Conclusion

Researchers conducting evaluative research of health systems in LICs sometimes find themselves in an ambiguous situation wherein their research results could improve health interventions, but the dissemination of these results could have negative consequences for the participants. For example, in this reflexive analysis, we have shown how having to obtain permission from high-level authorities to collect sensitive data in a few small facilities that local stakeholders knew well rendered it more difficult to preserve the confidentiality of participants during results dissemination. While this ethical issue may not be exclusive to health systems research in global health, we repeatedly found that it can be a challenge in this field. This issue may be relevant for any researcher working within a context of high inter-knowledge where people know each other and where it is difficult to separate the research setting from the results dissemination setting [14]. Given the above, what positions and actions should researchers take when faced with the conflicting imperatives of a) disseminating results to improve policies and practices and b) protecting individuals and groups at the local level? Researchers must develop and adopt strategies that enable them to respect their promise of confidentiality while effectively disseminating results that can sometimes be sensitive. Future research should examine the strategies that global health researchers from different methodological traditions are adopting in the field to reconcile both obligations. Moreover, research should attempt to better understand how ‘confidentiality’ is understood and operationalized as a concept in the sociopolitical and cultural contexts of these Western African countries. Going forward, reflections surrounding ethical issues in global health research should be deepened to better address how to manage competing ethical responsibilities while promoting valuable research uptake.

## Abbreviations

LICs: Low-income countries

## Footnotes

1 There are some exceptions regarding the participants’ willingness to be identified [7]. For example, in some forms of participatory action research, participants want their voices to be widely heard and insist that their identity be revealed.

2 The differences in characteristics between global health researchers and participants are partly related to the fact that most global health centres are located in high-income countries, that the field transcends national boundaries, and that it must reach across socio-economic boundaries to address health equity and disparities [16].
